# Surfaces and Air Bacteriology of Selected Wards at a Referral Hospital, Northwest Ethiopia: A Cross-Sectional Study

**DOI:** 10.1155/2018/6413179

**Published:** 2018-05-13

**Authors:** Hailu Getachew, Awoke Derbie, Daniel Mekonnen

**Affiliations:** ^1^Amhara Public Health Institute (APHI), Bahir Dar, Ethiopia; ^2^Department of Medical Microbiology, College of Medicine and Health Sciences, Bahir Dar University, Bahir Dar, Ethiopia

## Abstract

**Background:**

The hospital environment is a source of medically important pathogens that are mostly multidrug resistant (MDR) and posing a major therapeutic challenge. The aim of this study was to assess the surface and air bacteriology of selected wards at Felege Hiwot Referral Hospital (FHRH), Northwest Ethiopia.

**Methods:**

A cross-sectional study was carried out from 15th February to 30th April 2017. A total of 356 surface and air samples were collected from selected wards using 5% sheep blood agar (Oxoid, UK) and processed at FHRH microbiology laboratory following the standard bacteriological procedures. Pure isolates were tested against the recommended antibiotics using Kirby–Bauer disc diffusion methods, and the susceptibility profile was determined based on Clinical Laboratory Standards Institute (CLSI). Data were entered and analyzed using SPSS version 23 for Windows.

**Results:**

Of the total 356 samples processed, 274 were from surfaces and 82 were from air. Among these, 141 (39.6%) showed bacterial growth, yielding a total of 190 isolates. Gram-positive isolates were predominant at 81.6% (*n*=155), while the gram negatives were at 18.4% (*n*=35). The main isolates were coagulase negative staphylococci (*CoNs*), 44%, followed by *S. aureus*, 37.4%, and *Klebsiella species* at 11.6%. The bacterial load on surfaces and air was found beyond the standard limits. Besides, the antimicrobial susceptibility profile of the isolates showed that about 75% of the identified isolates were found resistant for two and more antimicrobial agents tested.

**Conclusions:**

This study showed high degree of bacterial load that is beyond the standard limits on both surfaces and air samples of the hospital. Furthermore, some 75% of the isolates were found multidrug resistant. Therefore, it is important to evaluate and strengthen the infection prevention practice of the hospital. Moreover, stakeholders should also reinforce actions to decrease the pressure of antimicrobial resistance in the studied area.

## 1. Background

Nosocomial infections (NIs) are infections acquired in a hospital or healthcare service unit that appear 48 hours or more after hospital admission or within 3 days after discharge [1]. The hand contact surfaces, floors, and air of the hospital environments are the main source of different pathogens that can cause NIs [2, 3]. About 5% to 10% of admitted patients to modern hospitals in the western countries acquire one or more NIs [4, 5]. In contrast, the magnitude of NIs is much higher in the developing countries due to different reasons [6] like poor ventilation system, high dusting, overcrowded setting, spread through sneezing and coughing, high movement of personnel, and suboptimal management of the hospital environment [7]. The hospital environment is the highest dissemination reservoir of pathogenic microbes which cause big challenges in the hospital environment, particularly in terms of NIs because it contains diverse population of microorganisms [7].

Microorganisms such as bacteria, fungi, and viruses can cause NIs. Reports showed that bacteria are much more important on this regard [8, 9]. The most common organisms usually associated with NIs are *S. aureus*, *CoNs*, *Pseudomonas aeruginosa*, *E. coli*, *Klebsiella* species, and proteus species that would source from patients, health personnel, attendants, contaminated instrument, and the environment [10–12]. Most strains of bacteria in the health service environments are multidrug resistant (MDR) [13]. The wide spread use of drugs, especially over or inappropriate use of antibiotics, has contributed to an increased incidence of antimicrobial resistant organisms, especially in developing countries [14–16].

Studies on bacteriological quality of wards of healthcare facilities in Ethiopia are scarce, and the few available ones reported unacceptably high bacterial load [13, 17]. Nationally, infection prevention guideline has been developed for healthcare facilities in Ethiopia. However, adherence of the healthcare providers to the protocol is quite limited [18] that could play a role for poor microbiological quality in different health facilities [19].

Based on our observation, in the present study setting, there were a number of health science students and high patient and patients' family trafficking in each ward of FHRH. Furthermore, there was poor restriction for entry of unauthorized individuals to access the different units of the hospital. Many wards were highly condensed and were not well ventilated. With this background and availability of quite limited data on the subject in the study area, this study was conducted to determine the degree of bacterial contamination and their antibiotic susceptibility profile from selected wards at Felege Hiwot Referral Hospital (FHRH) as part of infection prevention service auditing.

## 2. Materials and Methods

### 2.1. Study Setting, Design, and Period

We have conducted a hospital-based cross-sectional bacteriological study from 15 February to 30 April 2017 at FHRH, Bahir Dar, Northwest Ethiopia. Bahir Dar city is located about 565 km away from Addis Ababa, the capital of Ethiopia. FHRH was established in 1952 and was serving more than 10 million people of Bahir Dar and the surrounding zones and regions. The hospital had 13 wards, 430 beds, and about 531 health professionals during the period of data collection. The daily outpatient clients were more than 600. The hospital was also hosting medical and other health science students of Bahir Dar University and private colleges for practical attachments.

The authors had observational assessment in each ward during the sample collection period to assess the environmental cleanliness, number of occupants in each room, the use and type of disinfectants, the situation of ventilation, preparation of disinfectants, and frequency of cleaning. Based on our observation, all ward floors were cleaned with solution containing bleach three times a day. Dry sweeping was practiced before mopping which might suspend pathogens in the air. There was no mechanical ventilation system in any of the wards. Only the natural air circulates in the rooms which may increase the possibility of entrance of organisms from the outside environments. In all wards, we have observed no regular cleaning practice of hand contact surfaces such as walls, chairs, beds, intravenous (IV) stands, and stretchers. On the contrary, regular cleaning practice in all healthcare workers personal protective equipments was practiced. However, there was poor practice of removal and discarding of personal protective equipment prior to leaving the patient room.

### 2.2. Bacteriological Sampling and Culture

A total of 356 surface and air bacteriological samples were collected for analysis. Considering the number of patient flow and the safety of critically ill patients, the following hospital environments were included for sampling: Operation theater (OT), surgical wards, intensive care unit (ICU), neonatal intensive care unit (NICU), dialysis and obstetrics, maternity, and orthopedics. The rest areas of the hospital were excluded.

The air samples were collected two times per day: in the morning between 10 am and 11 am and in the afternoon between 1 pm and 2 pm, taking the consideration of high human trafficking in these time intervals. Samples were collected as per the standard protocol using the settle plate or passive air sampling method following the 1/1/1 schedule (on 90 mm diameter sterile petri dishes containing 5% sheep's blood agar left on the air for 1 hour, 1 meter above the floor, and 1 meter away from the wall) [20]. During air sampling procedure, sterile gloves, surgical masks, and protective gowns were used to prevent contamination of the agar plates. Plates were checked visually for any bacterial growth before it was used.

Similarly, sterile cotton swabs moistened with sterile normal saline were used to collect surface samples on 1 cm by 1 cm·area/cm^2^/surfaces such as the floor, walls, equipment, instruments, operation tables, sink, light switch, chairs, beds, patient cloths, door/locker handlers, trolley, stretchers, sinks/faucets, intravenous stands, and oxygen cylinder [21]. All type samples were labeled properly and transported to FHRH Microbiology laboratory within 30 minutes for microbiological analysis.

Both air and surfaces samples were inoculated on blood agar plates and incubated at 37°C for 18–24 hours. Identification of the isolates was done based on the standard microbiological procedures. Colony characteristics, gram reaction, and conventional different biochemical tests were used to identify the isolates [22]. Microbial concentration of air was expressed as interims of colony-forming units (CFUs) using colony counter, and the results were expressed in cfu/dm^2^/hr as described previously [23]. Similarly, swab culture result was expressed in colony-forming units using colony counter, and results were expressed in cfu/cm^2^ [21].

### 2.3. Antimicrobial Susceptibility Testing

Antimicrobial susceptibility profile of the isolates was performed based on the Kirby–Bauer agar disc diffusion method. The suspension of the identified test organism was prepared from similar colonies. The densities of suspension were determined by comparing with McFarland 0.5 Barium sulfate solutions [24]. A sterile swab dipped into the suspension of the isolate in broth and then speeded over the entire surface of the Muller–Hinton agar plate (Oxoid, LTD). Then, the antibiotic disks were placed on the surface of inoculated agar and incubated at 37°C for 18–24 hours. The diameters of the growth inhibition of discs were measured and interpreted as per the Clinical Laboratory Standards Institute (CLSI) guideline [25]. The drugs tested for both gram negative and gram positives were ciprofloxacin (5 *μ*g), gentamicin (10 *μ*g), tetracycline (30 *μ*g), cotrimoxazole (25 *μ*g), chloramphenicol (30 *μ*g), ceftriaxone (30 *μ*g), norfloxacillin (10 *µ*g), and Augmentin (30 *µ*g). Ampicillin (10 *µ*g) was tested only for gram negatives. In contrast, penicillin (10 IU), erythromycin (15 *μ*g), cefoxtin (30 *μ*g), doxycyclin (30 *µ*g), clindamycin (2 *µ*g), and clarithromycin (15 *µ*g) tested for gram positives [25].

### 2.4. Data Analysis

All data were entered, cleaned, and analyzed using Statistical Software Package for Social Sciences (SPSS) version 23 (SPSS Inc., Chicago, IL, USA) for Windows. Generated data were compiled and presented using descriptive statistics.

### 2.5. Quality Control

The reliability of the study findings was guaranteed through the implementation of standard quality control (QC) measures throughout the whole processes of the laboratory works. All culture plates were prepared according to the manufacturers' instructions. Control bacteria strains, like *Escherichia coli* (ATCC 25922), *S. aureus* (ATCC 25923), and *Pseudomonas aeruginosa* (ATCC 27853), were used to ensure the quality of culture plates and antimicrobial susceptibility testing discs [25].

### 2.6. Operational Definitions

- *Indoor air*: the air inside the rooms of the selected wards.
- *Settle plate or passive air sampling*: Petri dishes containing blood agar plates are left open to air for a given period of time. Microbes carried by inert particles fall onto the surface of the nutrient, with an average deposition rate of 0.46 cm/s being reported [20].
- *MDR bacteria*: those bacterial isolates that are resistant to two or more antimicrobial agents tested.

## 3. Results

### 3.1. Bacterial Profile of Surfaces and Air

**Figure 1:**
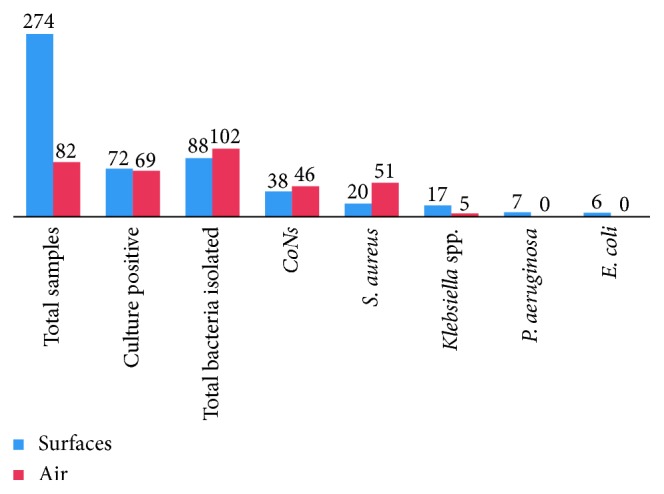
Types and frequency of bacterial isolates identified from the surface and of FHRH, 2017.

A total of 356 samples (274 surfaces and 82 air samples) were analyzed, of which some 141 (39.6%) showed bacterial growth yielding a total of 190 isolates. Mixed growth was reported on 42 (29.8%) samples. Gram-positive isolates were predominate at 155 (81.6%) followed by gram negatives, 35 (18.4%). Majority of the isolated bacteria at 102 (53.7%) were recovered from air, and the rest at 88 (46.3%) were from surfaces (Figure 1). The distribution of the isolates from surfaces includes door/locker handlers and floors (each account at *n*=12), linen and patient cloth (*n*=11), chairs (*n*=10), light switch (*n*=9), and sink (*n*=8).

Concerning the identified bacteria, the predominant isolates were coagulase negative staphylococci (*CoNs*) at 44%, followed by *S. aureus* at 37.4%, and *Klebsiella species* at 11.6%. *S. aureus* were isolated from the surgical ward and maternity ward at 19 (26.8%) each followed by orthopedics and the operation theatre at 12 (17%) each, and 18 (81.8%) of the *Klebsiella* spp. was also isolated in surgical wards.

When we look at the distribution of isolates from different wards, the highest bacterial growth was recovered from the surgical ward at 62 (32.6%) followed by maternity, orthopedics, and operation theatres with 49 (25.9%), 31 (16.3%), and 29 (15.3%), respectively. The least bacterial growth was documented in NICU, ICU, and dialysis rooms at 12 (6.3%), 5 (2.6%), and 2 (1%), respectively.

### 3.2. Bacterial Load from Air

The authors determined the degree of bacterial load from air of different wards based on the recommended approach. In terms of the distribution of wards, the highest numbers of bacteria isolates were identified from the surgical ward at 30.2% followed by maternity wards at 26.5%, orthopedics, and NICU with 9.8% each.

The profile of bacterial load of an open-air in terms of colony-forming units/dm^2^ is presented in Table 1. The highest load which was found beyond the standard limit at 721 cfu/dm^2^ and 619.3 cfu/dm^2^ was reported in the surgical and maternity wards, respectively. However, compared with the other wards, the bacterial load in the dialysis room was the least at 135.8 cfu/dm^2^. In the investigated rooms, the highest mean bacterial colony-forming units (CFUs) were recorded in the morning (10 : 00–11 : 00 am) at 482.8 cfu/dm^2^ (59.5%) compared with the afternoon (1 : 00–2 : 00 pm) at 329 cfu/dm^2^ (40.5%).

### 3.3. Bacterial Load from Surface

The mean aerobic colony count (ACC) from surfaces in the hospital was higher than the acceptable limits, at <5 cfu/cm^2^ [21]. The mean total aerobic colony counts from all surfaces in the investigated wards were at 31.5 cfu/cm^2^. The highest mean bacterial colony number was reported in surgical wards at 48.8 cfu/cm^2^ followed by maternity, orthopedics, NICU, and ICU at 45.9 cfu/cm^2^, 34.9 cfu/cm^2^, 27.5 cfu/cm^2^, and 16.5 cfu/cm^2^, respectively, and the least was in the OT at 14.8 cfu/cm^2^. No bacteria were isolated from the dialysis room.

### 3.4. Antimicrobial Resistance Profile of the Isolates

The gram-positive isolates, *CoNs*, showed high level of resistance against penicillin, clarithromycin, and erythromycin at 88%, 78.5%, and 70.2%, respectively. In contrast, these isolates showed low level of resistance to clindamycin, amoxicillin/clavulanic acid, and norfloxacillin at 17.2%, 19%, and 22.6%, respectively.

Similarly, *S. aureus* isolates also showed high level of resistance against penicillin, erythromycin, and clarithromycin at 84.5%, 75.5%, and 70%, respectively. Low level resistance at 16.3%, 22.5%, 22.5%, and 23.3% was documented against clindamycin, ciprofloxacin, amoxicillin/clavulanic, and Norfloxacillin, respectively.

On the other hand from gram-negative isolates, *E. coli* showed 100% resistance for ampicillin and cotrimoxazole. In contrast, lower level of resistance at 12.7% and 33.3% against ceftriaxone and norfloxacillin, respectively, was documented for *E. coli* (Table 2).

The overall drug resistance profile of the isolated bacteria showed that some 72 (46.6%) of gram-positive bacteria were resistant for five and more antimicrobial agents tested. Similarly, about 16 (45.7%) of gram-negative isolates were found resistant to five and more antimicrobial agents tested. About 5 of 190 isolates were found resistant to all of the antibiotics tested (Table 3).

## 4. Discussions

Different studies had reported that air and hand contact surfaces of the healthcare service units are contaminated by different pathogens which might serve as source of infections. This study was carried out to gain an insight into the distribution, frequency, bacterial load, and antimicrobial susceptibility profile of pathogens at the setting of FHRH, which is one of the busiest hospitals in Northwestern Ethiopia.

The aerobic culture results revealed that about 141 (39.6%) surfaces and air samples were found contaminated by various bacterial pathogens. This finding is relatively lower than other similar studies done in Ethiopia and abroad in Nigeria that reported bacterial growth at 52.9% and 65.7%, respectively [11, 27]. In the present study, about 81.6% of the isolates were gram positive which is in line with previous studies done in Ayder Hospital, Ethiopia, that reported 87.3% [16]. In contrast, lower distribution of gram positives at 43.1% was reported in Hawassa, Ethiopia [14]. The higher frequency of the gram positives might be due to the dry conditions of the hospital environment and transmission from skin, nasal, and boils of healthcare workers and patients as described previously [7, 28].

When we see the specific type of the isolates, *CoNs at* 84 (44.2%), *S. aureus* at 71 (37.4%) and *Klebsiella species at* 22 (11.6%) were the predominant. All of these are known nosocomial pathogens especially in surgical ward, OT & admitted immune-suppressed patients in hospital setting [12]. This result was found concurring with studies carried out in another parts of Ethiopia, Ayder and Hawassa University Hospitals [16, 29] but disagree with the one done in Mexico [30]. It was in surgical ward where the highest number of isolates recovered at 34.9% from air and surface samples compare with other selected wards which imply that the risk of contracting nosocomial infections in this ward would be higher.

From the total hospital air samples processed during the study period, about 69 (84.1%) showed bacterial growth. This entailed that numerous pathogenic bacteria could remain suspended in the air. Our finding is similar with a study done in Hawassa where the recovery rate from air was at 96.9% and Gondar University Hospital at 81.1% [11, 14]. The mean bacterial load in the air of the surgical ward, 721 cfu/dm^2^, and maternity ward, 619.3 cfu/dm^2^, was beyond the standards (250–450 cfu/dm^2^) set by Fisher et al. [26] and Pasquarella et al. [20]. However, similar findings were reported in the surgical ward at Jima University hospital at 463 cfu/dm^2^ [13]. In contrast, the reported bacterial load in ICU was at 246.9 cfu/dm^2^ which is in line with the standard, although different figures are presented by other studies done in Nigeria & Hawassa at 514 cfu/dm^2^ and 454.4 cfu/dm^2^, respectively [7, 15]. In the present study, the bacterial load of the OR (during active time) was at 249.4 cfu/dm^2^ which was three times higher than the standard limit that is indeed unacceptable. The possible explanation for the reported high load of mean aerobic bacterial counts could be due to poor ventilation and cleaning practices and high and unrestricted human trafficking, particularly medical/health science students who were attached in the hospital as part of their practical learning process. Comparable finding was reported on studies done in the other parts of Ethiopia [15, 16].

In the present study, the reported mean aerobic colony count from surface samples collected in the surgical ward, ICU, orthopedics, OT, maternity ward, dialysis, and NICU units was at 48.8 cfu/cm^2^, 16.5 cfu/cm^2^, 34.9 cfu/cm^2^, 14.6 cfu/cm^2^, 45.9 cfu/cm^2^, 0 cfu/cm^2^, and 27.5 cfu/cm^2^, respectively. This finding is beyond the acceptable limits set by Dancer, which states that the mean aerobic count from bacteriological culture of surface samples should be <5 cfu/cm^2^ [21]. The reported figure might add an increased risk of infection for patients in the studiedy hospital environment. In addition, the finding calls stakeholders to evaluate and strengthen the practice of infection prevention protocols strictly and to regularly monitor bacteriological quality of the hospital environment.

In this study, the authors tested the antimicrobial resistance profile of the isolates against commonly prescribed agents to highlight their up-to-date profile. Medically important bacteria are continuing posing a growing concern worldwide interim of their management choice. The wide spread use of drugs, especially over/inappropriate/use of antibiotics, unavailability of periodically updated guideline regarding the selection of drugs, and lack of routine microbiological technique to test the antimicrobial susceptibility profile of common agents share their great parts for antimicrobial resistance [11, 12, 14]. In the present study, the majority of gram-positive isolates showed resistance against most of the antibiotics tested. Comparable findings were reported on a study done in Jimma, in which >80% resistance was indicated among gram positives [31]. Some 25% of *S. aureus* isolates were found resistant for cefoxtin which indicates that methicillin-resistant *S. aureus* (MRSA) is ever increasing from time to time. Among gram-negative isolates, *E. coli* were found 100% resistant to ampicillin & cotrimoxazole each. This finding is similar with reports from Gondar and Addis Ababa that showed >80% resistance [11, 12].

In this study, more than 75% of the isolates were found multidrug resistant (MDR). This finding is comparable with reports from Hawassa (73.8%) and Nigeria (65.4%) [14, 32]. However, our finding is a bit different from a report by Tesfaye et al. from Ayder Hospital (36.5%) [16]. The resistance among various infectious agents to different antimicrobial drugs has emerged as a cause of public health threat all over the world at a terrifying rate, that really need urgent integrative intervention to curb the problem. Large amounts of antibiotics used for medical therapy, as well as for farm animals resulted in the selection of pathogenic bacteria resistant to multiple drugs (experts opinion).

Due to quite limited variables in this study, it was not possible to determine the associated factors that contribute for bacterial isolation and its load from the hospital environment.

## 5. Conclusions

The present study showed that surfaces and air in the different wards of the studied hospital were found contaminated with different types of bacteria. The bacterial load of surfaces and air were beyond the standard limits. The study also showed that there was an alarmingly high level of antimicrobial resistance for commonly prescribed drugs among isolates. Therefore, interventional strategies to scale up the practice of infection prevention in the hospital should be strengthened. Continuous surveillance and monitoring of the types and susceptibility patterns of nosocomial pathogens have to be periodically practiced. Furthermore, large-scale studies with sound sample size and design should be considered.

## Figures and Tables

**Table 1:** Mean bacterial counts from air sample at FHRH, 2017 [20, 26].

Site	Mean bacterial load Cfu/dm^2^/hr	Standard (cfu/dm^2^/hr)^∗^		
		Optimal	Acceptable	Unacceptable
Surgical ward	721 cfu/dm^2^	0–250	251–450	>450
OR (active)	294.4 cfu/dm^2^	0–60	61–90	>90
Maternity	619.3 cfu/dm^2^	NS	251–451	>451
NICU	292.8 cfu/dm^2^	NS	51–90	>90
ICU	246.9 cfu/dm^2^	0–250	251–450	>450
Orthopedics	580.2 cfu/dm^2^	NS	NS	NS
Dialysis	135.8 cfu/dm^2^	NS	NS	NS

^∗^Debatable whether the listed standards are commonly accepted; NS, no standard set.

**Table 2:** Distribution of antimicrobial resistance profile of bacterial pathogens isolated from surfaces and air at FHRH, 2017.

Antibiotics	Bacteria isolates and their antibiotic resistance pattern				
	*S. aureus* (*n*=71)	*CoNs* (*n*=84)	*Klebsiella* spp. (*n*=22)	*P. aeruginosa* (*n*=7)	*E. coli* (*n*=6)
Cotrimoxazole	35 (49.5%)	48 (57.7%)	8 (36.4%)	7 (100%)	6 (100%)
Chloramphenicol	20 (28%)	26 (31%)	7 (32%)	4 (57.2%)	4 (66.7%)
Gentamicin	19 (26.7%)	26 (30.5%)	9 (41%)	2 (28.6%)	4 (66.7%)
Tetracycline	29 (41%)	45 (53.4%)	7 (32%)	5 (71.6%)	4 (66.7%)
Cefoxtin	18 (25%)	24 (29%)	NA	NA	NA
Clindamycin	12 (16.3%)	14 (17.2%)	NA	NA	NA
Doxycyclin	17 (24.5%)	24 (29%)	NA	NA	NA
Erythromycin	54 (75.5%)	59 (70.2%)	NA	NA	NA
Clarithromycin	50 (70%)	66 (78.5%)	NA	NA	NA
Ciprofloxacin	16 (22.5%)	20 (24%)	5 (23%)	6 (85.7%)	4 (66.7%)
Norfloxacillin	17 (24%)	19 (22.6%)	4 (18.2%)	0 (0%)	2 (33.3%)
Augmentin	16 (22.5%)	16 (19%)	5 (23%)	IR	3 (50%)
Penicillin	60 (84.5%)	74 (88%)	NA	NA	NA
Ceftriaxone	23 (32.4%)	54 (64%)	9 (41%)	IR	1 (12.7%)
Ampicillin	NA	NA	12 (54.6%)	IR	6 (100%)

NA, not applicable; Augmentin, amoxicillin/clavulanic acid; IR, intrinsic resistance.

**Table 3:** Multidrug-resistant (MDR) profile of the isolates from surface and air of FHRH, 2017.

Types of spp.	Antibiogram profile					
	R0	R1	R2	R3	R4	≥ R5
Gram positive	**9 (5.8%)**	**18 (11.6%)**	**15 (9.7%)**	**27 (17.4%)**	**14 (9%)**	**72 (46.6%)**
*S. aureus*	8 (11.3%)	8 (11.3)	14 (19.7%)	11 (15.5%)	6 (8%)	24 (33.8%)
*CoNs*	1 (1.2%)	10 (11.9%)	1 (1.2%)	16 (19%)	8 (9.5%)	48 (57.1%)
Gram negative	**4 (11.4%)**	**9 (25.7%)**	**3 (8.6%)**	**2 (5.7%)**	**1 (2.9%)**	**16 (45.7%)**
*Klebsiella* spp.	4 (18.2%)	9 (40.9%)	1 (4.5%)	1 (4.5%)	0	7 (31.8%)
*E. coli*	0	0	2 (33.3%)	0	0	4 (66.7%)
*P. aeruginosa*	0	0	0	1 (14.3%)	1 (14.3%)	5 (71.4%)
Total	13 (9.8%)	28 (14.7%)	18 (9.5%)	29 (15.3%)	30 (15.8%)	88 (46.3%)

R0 = sensitive to all drugs, R1 = resistance to one drug, R2 = resistance to two drugs, R3 = resistance to three drugs, R4 = resistance to four drugs, and R5 = resistance to five drugs.

## Data Availability

All data generated during this study are included in this manuscript.

## Abbreviations

CFU: Colony-forming units
CLSI: Clinical Laboratory Standards Institute
FHRH: Felege Hiwot Referral Hospital
ICU: Intensive Care Unit
MDR: Multidrug resistant
NICU: Neonatal Intensive Care Unit
NIs: Nosocomial infections
OT: Operation Theatre
QC: Quality control
SPSS: Statistical Software Package for Social Sciences.
