# Corrigendum to “Surfaces and Air Bacteriology of Selected Wards at a Referral Hospital, Northwest Ethiopia: A Cross-Sectional Study”

**DOI:** 10.1155/2018/2190787

**Published:** 2018-07-29

**Authors:** Hailu Getachew, Awoke Derbie, Daniel Mekonnen

**Affiliations:** ^1^Amhara Public Health Institute (APHI), Bahir Dar, Ethiopia; ^2^Department of Medical Microbiology, College of Medicine and Health Sciences, Bahir Dar University, Bahir Dar, Ethiopia; ^3^Center for Innovative Drug Development & Therapeutic Trials for Africa (CDT-Africa), Addis Ababa University, Addis Ababa, Ethiopia

In the article titled “Surfaces and Air Bacteriology of Selected Wards at a Referral Hospital, Northwest Ethiopia: A Cross-Sectional Study” [1], there was a missing affiliation for the second author. The corrected authors' list and affiliations are shown above.
